# Development and Application of an Assay to Evaluate the Anti-Parasitic Effect of Humoral Responses against *Trypanosoma cruzi*

**DOI:** 10.3390/microorganisms11020241

**Published:** 2023-01-18

**Authors:** Nieves Martinez-Peinado, Juan Carlos Gabaldon-Figueira, Ignacio Martinez-Añon, Cristian Rodríguez-Gordo, Raquel Robleda-Castillo, Maria-Jesus Pinazo, Pascal Bigey, Joaquim Gascon, Julio Alonso-Padilla

**Affiliations:** 1 Barcelona Institute for Global Health (ISGlobal), Hospital Clínic—University of Barcelona, 08036 Barcelona, Spain; 2 Secció de Parasitologia, Departament de Biologia, Sanitat i Medi Ambient, Facultat de Farmàcia i Ciències de l’Alimentació, Universitat de Barcelona, 08007 Barcelona, Spain; 3 Laboratori de Referència Catalunya, 08820 Barcelona, Spain; 4 Drugs for Neglected Diseases initiative (DND*i*), 1202 Geneva, Switzerland; 5 CIBER de Enfermedades Infecciosas, Instituto de Salud Carlos III (CIBERINFEC, ISCIII), 28220 Madrid, Spain; 6 Université Paris Cité, CNRS, INSERM, UTCBS, F-75006 Paris, France; 7 Chimie ParisTech, PSL University, F-75005 Paris, France

**Keywords:** Chagas disease, *Trypanosoma cruzi*, humoral response, B-cell epitopes, ELISA, anti-parasitic assay

## Abstract

Mounting a balanced and robust humoral immune response is of utmost importance for reducing the infectivity of *Trypanosoma cruzi*. While the role of such a response in controlling the infection is well known, there is a lack of tools that can be used to quickly evaluate it. We developed a serum parasite inhibition assay (to evaluate changes in the parasite infection after exposing infective *T. cruzi* trypomastigotes to serum samples from infected patients). It is based on Vero cells as the hosts and the Tulahuen β-galactosidase parasite strain, genetically engineered to be quantifiable by spectrophotometry. In parallel, we developed an in-house ELISA to correlate the anti-*T. cruzi* antibody titres of the clinical samples with their observed anti-parasitic effect in the serum parasite inhibition assay. Serum samples from chronically *T. cruzi*-infected patients significantly inhibited parasite invasion in a titre-dependant manner, regardless of the patient’s clinical status, compared to samples from the non-infected controls. In addition, there was a clear correlation between the reactivity of the samples to the whole-parasite lysates by ELISA and the inhibitory effect. The results of this work confirm the previously described anti-parasitic effect of the serum of individuals exposed to *T. cruzi* and present a framework for its large-scale evaluation in further studies. The serum parasite inhibition assay represents a reproducible way to evaluate the intensity and anti-parasitic effect of humoral responses against *T. cruzi*, which could be applied to the evaluation of candidate antigens/epitopes in the design of Chagas disease vaccine candidates.

## 1. Introduction

Chagas disease affects over 6 million people around the world and is responsible for at least 10,000 deaths every year. The condition is caused by the protozoan parasite *Trypanosoma cruzi*, horizontally transmitted by hematophagous insects of the subfamily Triatominae. The infection can also be acquired orally through the ingestion of parasite- contaminated food and drink, or in a vector-independent manner through transplants and blood transfusions and vertically, from infected mothers to their offspring [1].

There are two drugs, benznidazole (BNZ) and nifurtimox (NFX), approved for the treatment of Chagas disease [2,3]. Despite both being highly effective in the early stages of infection, their efficacy is reduced in the chronic stage of the disease, when most cases are diagnosed. Regrettably, the development of new drugs is hindered by uncertainties regarding the pathogenic mechanisms through which the parasite causes cardiac conductivity disorders, heart failure, and the dilation of different segments of the digestive tract. These manifestations appear in approximately 30% of the chronically infected patients, compromising their life and eventually leading to death if unattended [4,5].

*T. cruzi* is a genetically diverse parasite, being classified into seven major lineages known as discrete typing units (DTUs): TcI–TcVI, and TcBat. Different DTUs are associated with distinct ecological niches, clinical signs, and different levels of susceptibility to drugs [6,7]. Such biological diversity is a challenge for the development of alternative chemotherapies and vaccines [8,9,10], which are also hindered by the parasite’s biological complexity. In mammals, its lifecycle involves an extracellular motile stage, the trypomastigotes, which disseminate the infection, and an intracellular obliged stage, the replicative amastigotes, in which the occurrence of dormant forms has been described [11], making an adequate cellular response essential for controlling the infection. On the other hand, initial humoral responses against *T. cruzi* are generally polyclonal and inefficient, delaying the development of a specific, neutralizing response [12]. Such a response, nonetheless, develops later in the course of infection and is important for reducing parasitemia in the chronic stages, as evidenced by the lethal effect of B-cell depletion in murine models of infection [13].

The inhibitory effect of specific antibodies targeting the surface antigens of *T. cruzi* on its in vitro development has been previously described [14,15], but the availability of a standardized, scalable, and adaptable assay that can be used to quantitatively asses the humoral response against specific *T. cruzi* antigens is highly desirable, given its potential use in the preclinical evaluation of vaccine candidates, some of which include B-cell epitopes [10,16]. In this paper, we describe an assay that can be used to evaluate the anti-parasitic effect of the humoral response against *T. cruzi* infection, comparing the capacity of serum from infected individuals to inhibit parasite cell invasion in vitro with that of non-infected control subjects of the same geographic origin. In the future, we expect that this assay could be applied to the study of the anti-infective capacity of sera obtained upon preclinical immunization with different parasite antigens or epitopes so that it may contribute to the design of the humoral components of anti-*T. cruzi* vaccine candidates.

## 2. Materials and Methods

### 2.1. Ethics

The serum sample collection and manipulation were performed following protocols approved by the Clinical Research Ethics Committee of the Hospital Clinic of Barcelona (HCB/2013/8031 (R130531-141)) and were compliant with the principles of the Declaration of Helsinki.

### 2.2. Sample Collection

Serum samples were collected from patients attending the Service of Tropical Medicine at the hospital clinic. The standard testing for *T. cruzi* infection involved two serological assays based on different antigen sets, in agreement with the WHO-PAHO recommendations [3]. All subjects were tested with a commercial enzyme-linked immunosorbent assay (ELISA) kit (Vircell Chagas ELISA IgG + IgM; Granada, Spain) and a chemiluminescent microparticle immunoassay (CMIA; ARCHITECT Chagas^®^, Abbot; Wiesbaden, Germany) and classified as positive or negative, accordingly [3]. All the samples from *T. cruzi*-infected individuals were from chronically infected adults. The control samples were from non-infected subjects from the same geographic region. All the serum samples were diluted 1:1 in glycerol and stored at −80 °C until needed.

### 2.3. Cell and Parasite Cultures

LLC-MK2 (Rhesus monkey kidney epithelial cells) and Vero (African green monkey kidney epithelial cells) cells were cultivated as described previously [17]. Parasites from the Tulahuen strain (DTU VI), engineered to express the *E. coli* β-galactosidase gene (kindly provided by Prof. Frederick Buckner, University of Washington, Seattle, WA, USA) [18] were used in all the experiments. The *T. cruzi* parasites were maintained in culture by infecting the LLC-MK2 cells and purified as described previously [17]. The cultures were maintained in Dulbecco’s modified Eagle medium (DMEM) enriched with 2% (parasite cultures) or 10% fetal bovine serum (Vero and LLC-MK2 cell cultures). All media were supplemented with 1% penicillin-streptomycin (PS) [17].

### 2.4. Design and Validation of the Serum Parasite Inhibition Assay

To evaluate the anti-parasitic effect of the serum, 50,000 Vero cells (50 µL of a 1 × 10^6^ cells/mL solution) were seeded in wells already containing 135 μL of assay medium (phenol-red-free DMEM supplemented with 2% FBS, 1% penicillin-streptomycin-glutamine, Na-pyruvate 1 mM, and 4-(2-hydroxyethyl)-1-piperazineethanesulfonic acid (HEPES) 25 mM) on 96-well tissue culture plates (SPL Life Sciences Co., Pocheon-si, Republic of Korea). The cells were incubated for two hours at 37 °C to enable attachment and monolayer formation.

In the meantime, the purified *T. cruzi* trypomastigotes were exposed to serially diluted serum samples from either *T. cruzi*-infected subjects or non-infected controls on a 96-well U-bottom plate (SPL Life Sciences Co., Pocheon-si, Republic of Korea). For this, a ¼ dilution of each 1:1 serum–glycerol (vol.:vol.) sample was prepared in the first column of the plate and serially diluted by ½ four times. After this, 20 μL of a 1 × 10^7^ trypomastigotes/mL solution was added to each well for a final volume of 60 µL per U-well. The dilution range of the sera was from 1/12 to 1/96, which guaranteed a final glycerol concentration below the limit of toxicity observed for Vero cells and the parasite (see below).

Upon incubation for 1 h at 37 °C on the U-bottom plate, 15 µL of the serum-trypomastigote mixture (~50,000 trypomastigotes) were transferred to the corresponding wells of the assay plates containing Vero cell monolayers, leading to a multiplicity of infection (MOI) = 1 and a final volume per well of 200 µL. Infection with the serum-trypomastigote mixture or serum-treated trypomastigotes alone did not have a significant effect on the results (data not shown). The assay plates were incubated overnight at 37 °C before washing off the excess trypomastigotes and sera with PBS. Finally, 200 μL/well of fresh assay medium were added, and the plates were incubated at 37 °C for another 72 h. The inhibition of parasite invasion into the cells was inversely proportional to its presence and growth in the tissue cultures, which we measured by reading the beta-galactosidase activity of the parasites by adding 50 μL per well of a PBS solution containing 0.25% NP40 and 500 μM of chlorophenol red-β-D- galactopyranoside (CPRG) [17]. 

The results were normalized to the controls’ maximum parasite growth (or no inhibition, represented by wells containing only Vero cells and non-exposed *T. cruzi* trypomastigotes). Additionally, a pooled sample of sera from subjects infected with *T. cruzi* was used as a positive inhibition control (positive pool), and a pooled sample of sera from non-infected subjects was used as a negative inhibition control (negative pool). These controls were used to test the statistical robustness of the assay, as described below. An extra inhibition control including serial dilutions of the standard drug BNZ was included to evaluate the consistency of the assay results. All the conditions were tested in at least two biological replicates with three technical replicates each. The results are provided as means ± standard deviation (SD). 

### 2.5. Determination of Glycerol Toxicity

To evaluate the effect of glycerol (in which the serum samples were stored) on the parasite’s development, we followed the same steps as those described above using PBS-diluted concentrations of glycerol ranging from 10% to 0.0048% instead of serum. On the other hand, we specifically evaluated the effect of glycerol on the host Vero cells, following a similar procedure without adding parasites. In this case, the readout was performed by recording the fluorescence intensity upon the addition of a solution containing 10% AlamarBlue^TM^ to assess the Vero cells’ viability [17].

### 2.6. In-House ELISA to Detect Anti-T. cruzi Antibodies

To correlate the capacity of the serum samples to inhibit parasite invasion in the presence of antibodies against the parasite antigens, we performed an in house-ELISA, relying on whole-parasite lysates as the antigen. 

The amastigote and trypomastigote forms were purified from the infected LLC-MK2 cultures. In brief, to obtain the trypomastigotes, supernatants were centrifuged at 2500 rpm for 10 min and left for 2 h at 37 °C to allow the trypomastigotes to swim out from the pellets. Amastigotes were obtained from the remaining pellets. Amastigotes and trypomastigotes were counted in Neubauer chambers before being frozen at −80 °C to form the lysates. The protein concentration was quantified using a commercial bicinchoninic acid assay (BCA, Thermo Scientific, Waltham, MA, USA).

For the coating, we mixed identical masses of trypomastigotes and amastigotes in carbonate-bicarbonate buffer (pH = 9.6) and used it to coat 96-well ELISA plates (Nunc MaxiSorp™ flat-bottom; Thermo Fischer Scientific, Waltham, MA, USA) overnight at 4 °C with 0.2 µg of antigen per well (1:1 mixture of trypomastigotes and amastigotes) (Figure S1). The plates were washed thrice with phosphate buffered saline–Tween 0.05% (PBST) and subsequently blocked for 1 h at 37 °C by adding 100 µL/well of a blocking solution formed of PBS and bovine serum albumin (BSA) at 3%. Another washing step was performed before adding 50 µL per well of the PBS-BSA 3% solution containing 1/640 serum dilutions that incubated for 1 h at 37 °C (Figure S1). The plates were washed 3× before incorporating the secondary antibody: 50 µL/well of goat anti-human IgG coupled with horseradish peroxidase (HRP) at 1/4000 dilution in PBS-BSA 3% for 1 h at 37 °C (Figure S1). Upon the final 5× PBST wash, the HRP activity was revealed with a 3,3′,5,5′-tetramethylbenzidine substrate (TMB, 50 µL/well; BD Biosciences, San José, CA, USA) after a 15 min incubation period in the dark at room temperature. The reaction was stopped with 25 µL per well of H_2_SO_4_ 3N, and the absorbance was read in the bi-chromatic mode at 450 and 620 nm using an Epoch Microplate Spectrophotometer (BioTek, Winooski, VT, USA).

### 2.7. Data Analysis

The statistical significance of the differences in parasite invasion between the wells treated with serum from subjects in different clinical groups (infected asymptomatic, infected symptomatic, or uninfected) was determined using a pair-wise Wilcoxon rank sum test adjusted for Bonferroni correction. Negative values of cell invasion inhibition were treated as no inhibition and transformed to zeroes so as to facilitate their graphical representation. In addition, the inhibition of the parasite invasion and antibody titres of individual samples were compared using linear regression. The performance of the serum parasite inhibition assay was evaluated by comparing the absorbance of the wells containing parasites exposed to pooled sera from the infected and non-infected subjects. These values were used to calculate the assay statistical robustness by means of the *Z*′ parameter [19], according to the following equation, in which values above 0.5 indicate a good capacity to discriminate between two different groups:

(1) $$Z^{\prime }=1-\frac{\left(3\sigma_{c+}+\sigma_{c-}\right)}{{µ}_{c+}-{µ}_{c-}}$$

where c+ indicates the positive controls, c− indicates the negative controls, σ indicates the standard deviation in the absorbance readings, and µ represents the mean absorbance.

The intensity of the humoral response mounted against the *T. cruzi* antigens was determined by calculating the ratio between the absorbance of the samples from subjects diagnosed with *T. cruzi* infection and the mean absorbance of the non-infected controls in the individual rounds of the previously described in-house ELISA (P/N ratio). The P/N ratios of individual samples were used to plot a receiving operating characteristic (ROC) curve (Figure S2), in turn used to determine the accuracy of the test and to select a cut-off for the P/N ratio in order to calculate the in-house ELISA sensitivity and specificity parameters in comparison to the commercial diagnostic kits used by the hospital (Table S1). All statistical analyses were performed using GraphPad Prism version 7.0 (GraphPad Software, San Diego, CA, USA) or RStudio (RStudio Team, 2022. RStudio: Integrated Development Environment for R. RStudio, PBC, Boston, MA, USA).

## 3. Results

The inhibitory capacity of 104 samples was evaluated using the serum parasite inhibition assay. Of these, 45 were from subjects previously diagnosed with Chagas disease, while 59 were from non-infected controls (Table 1). A total of 75 samples (72.1%) were obtained from women, and the median age of the study cohort was 42 years (IQR: 37–52, Table 1). Out of the 45 infected subjects, 39 (86.7%) were in the asymptomatic indeterminate stage, while 6 (13.3%) presented clinical symptoms characteristic of the chronic disease stage. Four (11.1%) participants presented with cardiological manifestations, one (2.2%) presented with digestive abnormalities, and one (2.2%) presented with alterations in both systems. Most of the samples (94/104, 90.4%) were from Bolivian participants. A full description of the study cohort is presented in the Table S2.

### 3.1. Anti-Parasitic Effect of Serum from Subjects Infected with T. cruzi

The viability of both the parasite and host cells was affected by glycerol concentrations above 0.625% per well, and therefore, the 1:1 glycerol-mixed serum samples in the serum parasite inhibition assay were diluted, so that all the wells presented a final glycerol concentration below this threshold (Figure S3).

**Figure 1:**
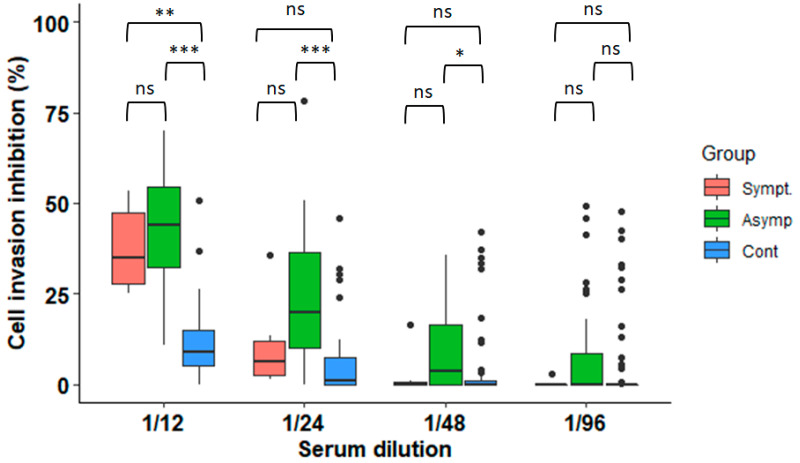
Inhibition of cell invasion after incubation with serum samples from non-infected controls and subjects infected with *T. cruzi*. (N = 104 samples; pairwise Wilcoxon test with Bonferroni correction: ***, *p* < 0.0001; **, *p* < 0.001, * *p* < 0.01, ns = non-significant).

Parasite invasion was inhibited in the wells containing parasites exposed to serum samples from the *T. cruzi*-infected controls (median inhibition at dilution 1/12 = 44% (IQR = 29.9–53.5%)) (Figure 1). In comparison, the parasites exposed to serum from the uninfected (negative) subjects at the same dilution showed a median inhibition of 8.94% (IQR = 5.12–15%, *p* < 0.0001). Moreover, the inhibitory effect was lower in the serum from the subjects with symptomatic infection (median inhibition = 34.8%) than in the asymptomatics (44.1%), but this difference was not statistically significant (Figure 1). Likewise, the median inhibition was higher in the samples from the infected male participants (25%, IQR: 10.5–50.4%) compared to the females (16%, IQR: 7.6–34.6%), but this difference was also not significant in any clinical group (*p* = 0.1 for the asymptomatics, *p* = 1 for the symptomatics, and *p* = 0.62 for the non-infected controls; Figure S4).

The invasion inhibition remained higher in the asymptomatically infected participants compared to the controls at dilutions 1/24 (*p* < 0.0001) and 1/48 (*p* = 0.0012) (Figure 1). The microscopic observation of the wells with serum from the *T. cruzi*-infected subjects revealed the agglutination of the trypomastigotes, suggesting that this phenomenon might have precluded the trypomastigotes from infecting the host cells (Figure S5).

### 3.2. The Serum Parasite Inhibition Assay Is Statistically Robust

**Figure 2:**
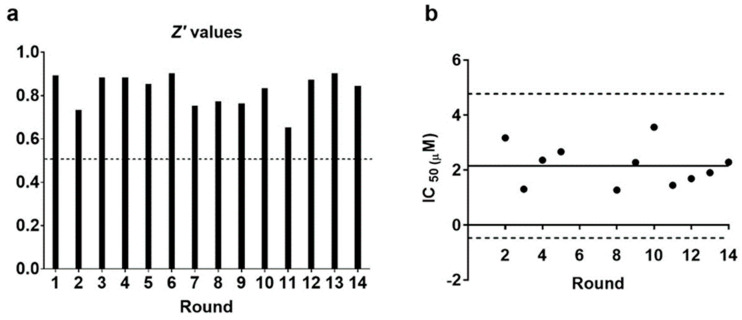
Statistical robustness of the serum parasite inhibition assay. (**a**) Z′ values and (**b**) IC_50_ values for BNZ obtained in different assay runs. The dashed line in (**a**) marks the 0.5 threshold, while the dotted lines in panel B represent ±3SD from the mean (continuous line).

The average Z′ value obtained after 14 rounds was 0.79 (SD = 0.15). Furthermore, it remained above the 0.5 threshold in all the rounds, indicating a good statistical robustness in differentiating the antiparasitic effect of the positive sera from the negative controls (Figure 2a). Additionally, the average IC_50_ for the BNZ controls in these assays was 2.71 µM (SD = 0.71), and the values derived from individual rounds remained similar and within a range of ±3SD, indicating that the assays were carried out consistently and yielded comparable results (Figure 2b). Altogether, these results confirm that the observed variation in absorbance was caused by the different capacities of the serum samples to inhibit parasite invasion.

### 3.3. Inhibition of Parasite Invasion Is Associated with Higher Anti-T. cruzi Antibody Titres

**Figure 3:**
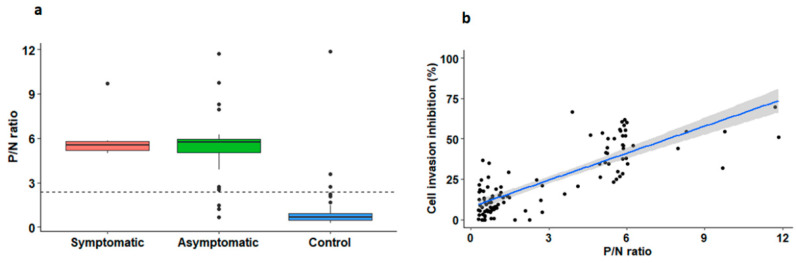
Inhibition of parasite cell invasion is associated with a stronger humoral response against *T. cruzi*. (**a**) Box plot of P/N ratios of the non-infected controls and *T. cruzi*-infected subjects with chronic manifestations of disease or in the indeterminate asymptomatic stage. (**b**) Inhibition of cell invasion in relation to P/N ratios obtained in the in-house ELISA. The dotted line in (**a**) represents the P/N positivity cut-off value for the in-house ELISA of 2.4, according to the ROC calculation. The blue line in (**b**) is the regression line and the gray area, the CI95% of the linear regression.

To further investigate whether the anti-parasitic effect was associated with a stronger humoral response against *T. cruzi*, we measured the serological reactivity of the samples to the whole-parasite lysates using an in-house ELISA. The participants infected with *T. cruzi* presented higher P/N ratios regardless of their clinical status (median P/N ratio of the infected participants: 5.72, IQR = 5.05–5.91, compared to 0.68, IQR = 0.46–0.94 for the controls, *p* < 0.0001, Figure 3a).

Remarkably, the sera from the subjects with higher P/N ratios in the ELISA inhibited parasite invasion more effectively (R^2^ = 0.66, *p* < 0.0001; Figure 3b). This result suggests that the inhibitory effect observed is partially explained by an enhanced humoral response against *T. cruzi* during the course of a natural chronic infection.

## 4. Discussion

Defining the in vitro correlates of protection against *T. cruzi* infection could greatly contribute to efforts to save developmental costs in the study of Chagas disease vaccine candidates [16]. In this regard, the availability of an assay that is able to measure the effectivity of the antibody response mounted against the infection in pre-clinical or clinical samples would be very valuable [20].

The current diagnostic tests, such as Vircell^TM^ ELISA or Abbot^TM^ CMIA, are based on a handful of recombinant antigens selected to yield the optimal sensitivity and specificity outputs. However, high antibody titres against these or other antigens might not necessarily correlate with protection, given the wide array of antigens to which the immune system is exposed throughout the course of a chronic *T. cruzi* infection and the parasite’s intrinsic genetic variability [20,21]. For instance, an intense polyclonal B-cell response, characterized by the production of high titers of antibodies with low parasite specificity, occurs during the initial stages of infection, without offering any protection [12,13]. Therefore, a careful evaluation of the anti-parasite effect of responses against any group of antigens is essential before their incorporation into vaccine candidates that will be evaluated in vivo. For this purpose, we developed an assay that can identify the inhibitory potential of human serum samples from chronically infected subjects in vitro, which could easily be adapted to include samples from pre-clinical animal models of infection. Traditionally, the efficacy or potency of prophylactic antigens has been measured by challenge studies, which involve the exposure of healthy animals to the pathogen. These studies have some limitations, such as the substantial number of animals required, the associated ethics concerns, and their cost. An initial evaluation of the inhibitory capacity of new antigens of interest through the described assay or similar assays may reduce the number of animals used in the preclinical evaluation of future vaccine candidates. 

Using a two-step process in which the infective trypomastigotes were exposed to either serum from *T. cruzi*-infected subjects or serum from non-infected subjects, we were able to demonstrate the inhibitory effects of the former on parasite invasion and growth in vitro. The fact that high serum dilutions failed to produce any observable inhibition of parasite invasion, as well as the trypomastigotes’ intrinsic resistance to the complement lysis [12], suggested that the observed effect was at least partially mediated by specific antibodies developed in the chronic stages of infection. This is in accordance with previous studies that have already described this phenomenon [14,15] and with the observed correlation between the P/N ratios in our in-house ELISA and lower parasite invasion. Anti-alpha-galactosyl antibodies, known to induce the agglutination of cell-derived trypomastigotes, a feature also observed in these experiments, are likely involved in this inhibitory effect [22]. On the other hand, Hernandez and co-authors found that serum from patients with Chagas disease that had been depleted of the complement system and specific antibodies had an anti-proliferative effect on *T. cruzi* epimastigotes. Similarly, during the setting up of the assay, we did not observe any differences in the ability to inhibit parasite cell invasion between the complement-depleted serum (heat inactivation at 56 ºC for 30 min) and the whole serum (data not shown). This suggests that the complement system did not contribute to the inhibition of the invasion, an observation explained by the several strategies of evasion that *T. cruzi* presents against the complement [23]. The data of Hernandez and co-authors support the idea that an enhanced production of reactive oxygen species in chronically infected patients might contribute to the observed anti-parasitic effects of their sera [24]. It cannot be discarded that such a phenomenon, as well as other innate response factors, might play a certain role in the output of the currently described parasite inhibition assay. However, the correlation observed between the higher antibody titres in the ELISA and the reduction in parasite development indicates that specific antibodies are most likely involved in the antiparasitic effects reported. 

Importantly, our methodology allows for the simultaneous processing of several samples, and the use of *T. cruzi* strains modified to be readily detectable by spectrophotometry greatly facilitates the assay readout. This is a significant difference compared to the similar assays currently used for the evaluation of humoral responses against *T. cruzi* in animal models [25,26,27]. Therefore, in addition to confirming the well-known anti-parasitic effect of serum from chronically infected subjects, our results provide a new tool that is highly applicable to different research contexts. 

A limitation that we faced during the setting up of the assay was the fact that the available serum samples in our collection were mixed with glycerol prior to storage for improved preservation. This restricted the range dilution of the sera to which the parasites could be exposed in the assay. Likewise, we found that the mixture of serum samples with glycerol can interfere with some of the biological assays to which those samples might be subjected. Therefore, it is advisable to store samples without glycerol if they are to be used in these invasion inhibition assays. Another limitation is that we used *T. cruzi* parasites from DTU VI and samples mostly from Bolivian patients, in whom this DTU is not the most prevalent [28]. Comparative genomics have shown the impressive diversity of genes encoding surface antigens, which play important roles in *T. cruzi* biology, infection, and the induction of the immune response [29]. Thus, it would be interesting to evaluate these serum samples against a panel of different *T. cruzi* DTUs in further experiments, as well as Chagas disease serological collections of other geographical origins. It must also be noted that the assay is technically complex and requires handling high concentrations of live, infective parasites, limiting its application to highly trained lab personnel.

Despite this, the serum parasite inhibition assay represents an efficient and standardized method that can be used to assess the anti-parasitic effects of antibodies raised against different *T. cruzi* antigens in preclinical and clinical settings. Similarly, our in-house ELISA can be readily adapted to detect antibodies against specific antigens of interest, coating the assay plates with such proteins rather than the whole-parasite lysates used in this work. In the future, this approach could be used to correlate the concentrations of specific antibodies with their inhibitory effects on parasite growth, an important step towards the identification of correlates of protection, or to validate the usefulness of putative antigens identified through bioinformatic approaches.

## Figures and Tables

**Table 1:** Study participant characteristics.

Sex	
Male (%)	29 (27.9%)
Female (%)	75 (72.1%)
**Median age (IQR)**	42.5 (36.7–52)
**Nationality**	
Bolivia (%)	94 (90.4%)
Paraguay (%)	2 (1.9%)
Argentina (%)	1 (1%)
Brazil (%)	1 (1%)
Peru (%)	1 (1%)
Ecuador (%)	1 (1%)
Spain (%)	1 (1%)
Unknown (%)	3 (2.9%)
**Clinical manifestations**	
Cardiovascular (%)	4 (3.8%)
Digestive (%)	1 (1%)
Cardiovascular and digestive (%)	1 (1%)
Indeterminate stage (asymptomatic) (%)	39 (37.5%)
**Total infected**	45
**Total non-infected**	59
**Total**	104

## Data Availability

The dataset supporting the conclusions of this article is included within the article and its additional files.

## Supplementary Materials

The following supporting information can be downloaded at https://www.mdpi.com/article/10.3390/microorganisms11020241/s1: Figure S1. Setup of the in-house ELISA test; Figure S2. Receiving operating characteristic (ROC) analysis of the performance of the in-house ELISA; Figure S3. Evaluation of the glycerol toxicity for the two cell types used in the serum parasite inhibition assay; Figure S4. Inhibition of parasite invasion after incubation with serum samples from female and male participants at dilution 1/12; Figure S5. Effect of serum incubation on trypomastigotes; Table S1. Performance of the in-house ELISA compared to commercial diagnostic kits used by the laboratory of the Hospital Clinic of Barcelona; Table S2. Master table for the in-house ELISA and parasite cell invasion inhibition assays.
