# Supplementary material for: Development and Application of an Assay to Evaluate the Anti-Parasitic Effect of Humoral Responses against Trypanosoma cruzi
Source: Microorganisms. 2023 Jan 18;11(2):241. doi: 10.3390/microorganisms11020241 (PMC9966445; doi:10.3390/microorganisms11020241)
Supplement: Supplementary file 1 [file microorganisms-11-00241-s001.zip › microorganisms-2106119-supplementary-materials.pdf]

microorganisms

Article

Antisera: An Assay to Evaluate the Anti-parasitic Effect of Hu-Moral Responses Against *Trypanosoma cruzi*

Nieves Martinez-Peinado [1,2,*], Juan Carlos Gabaldon-Figueira [1], Ignacio Martinez-Añon [1], Cristian Rodríguez-Gordo [3], Raquel Robleda-Castillo [1], Maria-Jesus Pinazo [1,4,5], Pascal Bigey [6,7], Joaquim Gascon [1,5] and Julio Alonso-Padilla [1,5,*].

1 Barcelona Institute for Global Health (ISGlobal), Hospital Clínic-University of Barcelona, Barcelona, Spain
2 Secció de Parasitologia, Departament de Biologia, Sanitat i Medi Ambient, Facultat de Farmàcia i Ciències de l'Alimentació, Universitat de Barcelona, Barcelona, Spain
3 Laboratori de Referència Catalunya, Prat de Llobregat (El), Barcelona, Spain
4 Drugs for Neglected Diseases initiative (DND*i*), Switzerland
5 CIBERINFEC, ISCIII—CIBER de Enfermedades Infecciosas, Instituto de Salud Carlos III, Madrid, Spain
6 CNRS, INSERM, UTCBS, Université Paris Cité, F-75006 Paris, France
7 Chimie ParisTech, PSL University, F-75005 Paris, France
* Correspondence: nieves.martinez@isglobal.org (N.M.-P.); julio.a.padilla@isglobal.org (J.A.-P.); Tel.: 0034-932275400 (ext. 4284)

Supplementary Materials

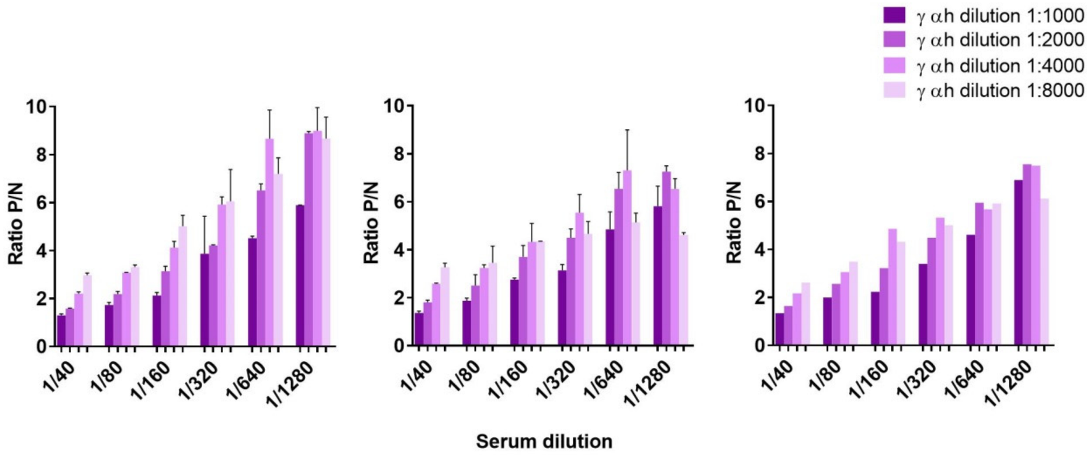

Figure S1. Set-up of the in-house ELISA test. Absorbance windows under different assay conditions using whole lysates of: (a) amastigotes; (b) trypomastigotes; or (c) amastigote/trypomastigote mix, as coating antigens. Colors represent different dilutions of the human γ-gαh secondary IgG antibody.

A checkerboard set-up was used to determine the optimal antigen concentration to be used in the in-house ELISA. Similarly, optimal concentrations of positive and negative pooled human serum (test) samples, and secondary goat anti-human (γ-gαh) IgG coupled to horseradish peroxidase (Jackson ImmunoResearch, Philadelphia, USA) were selected by testing serial dilutions ranging 1/40 – 1/1280, and 1/1000 – 1/8000, respectively.

The largest absorbance window between pooled positive and negative samples was observed using an antigen concentration of 0.2 μg per well (1:1 mixture of trypomastigotes and amastigotes), a 1/640 serum dilution, and a 1/4000 γ-gαh IgG dilution (Figure S1). Thereafter, the performance of the in-house ELISA under these conditions was evaluated by measuring the reactivity of individual serum samples from confirmed *T. cruzi*-infected and non-infected controls.

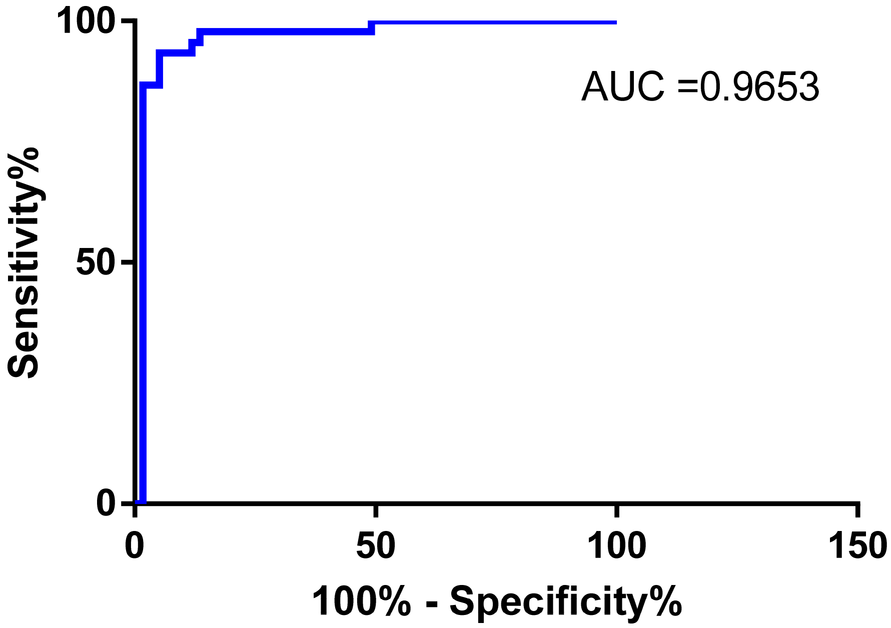

Figure S2. Receiving Operating Characteristics (ROC) analysis of the performance of the in-house ELISA. The sensitivity and specificity of the in-house ELISA was calculated for different P/N thresholds, and compared to the diagnosis provided by the Service of Tropical Medicine of the Hospital Clinic, obtained after using two different commercial kits (gold standard).

A ROC analysis revealed an area under the curve (AUC) of 0.965 (CI95%: 0.925 - 1.00; Figure S2). A cut-off value for the P/N ratio of 2.4 provided the highest sensitivity (93.3%; CI95%: 81.7 - 98.6) and specificity (94.9%; CI95%: 85.9 - 98.9), in comparison to the commercial ELISA and CMIA kits used by the Hospital Clinic of Barcelona.

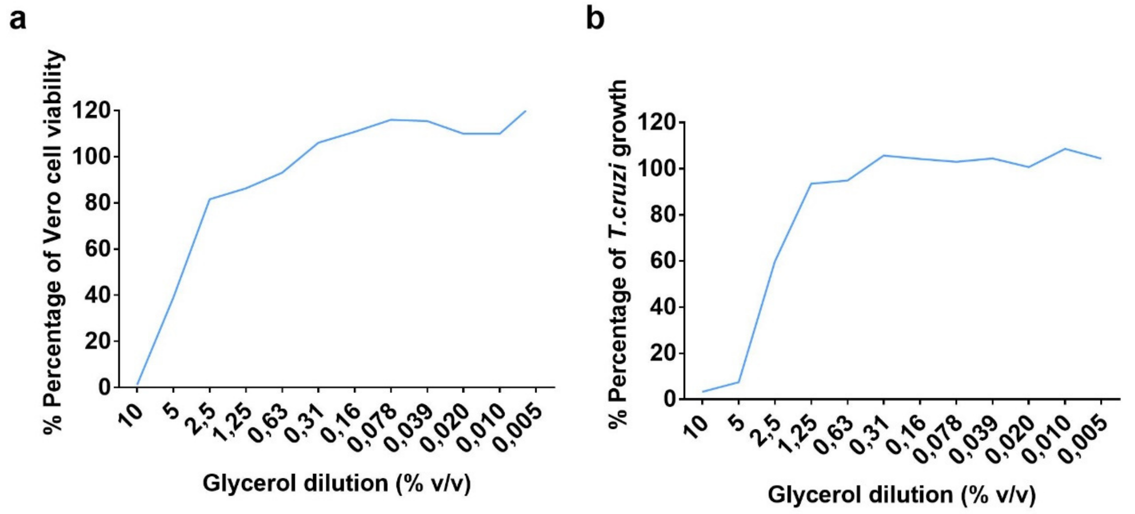

Figure S3. Evaluation of glycerol toxicity over the two cell types used in the antisera assay. (a) Uninfected Vero cells; and (b) *T. cruzi*-infected Vero cells cultures.

We observed that concentrations of glycerol above 0.625% per well were toxic to Vero cells and thus capable to interfering with the in vitro growth of *T. cruzi,* with host cells viability being markedly supressed at concentrations above 5%.

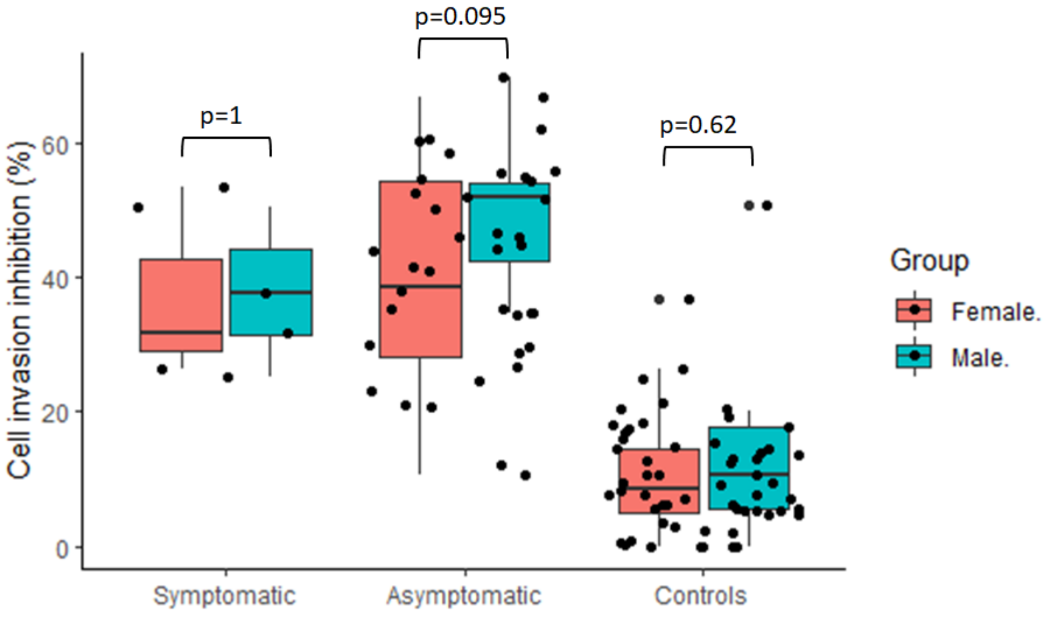

Figure S4. Inhibition of parasite invasion after incubation with serum samples from female and male participants at dilution 1/12. No statistically significant differences were observed between male and females in any clinical groups.

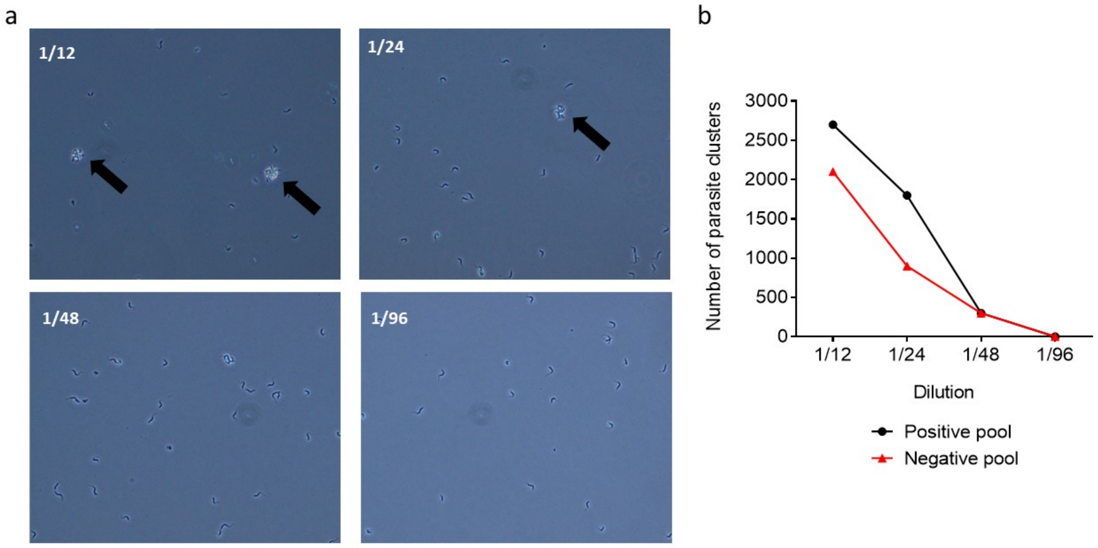

Figure S5. Effect of serum incubation on trypomastigotes. Representative images of trypomastigotes after incubating for 1 h at 37 ºC with positive sera at dilutions 1/12 to 1/96 (a); and the corresponding number of clusters of trypomastigotes (b). Pictures were taken at a 400× magnification with a microscope Olympus IX-51 (Hamburg, Germany). Notice clumping of parasites in panel a (arrowheads).

Table S1. Performance of the in-house ELISA compared to commercial diagnostic kits used by the laboratory of the Hospital Clinic of Barcelona.

		In-house ELISA		
		**Positive**	**Negative**	**Total**
**ELISA/CMIA Hospital Clinic**	**Positive**	42	3	45
	**Negative**	3	56	59
	**Total**	45	59	104

Table S2. Master table for the in-house ELISA and parasite cell invasion inhibition assays.

Available in the additional excel file.
